# Subliminally Perceived Odours Modulate Female Intrasexual Competition: An Eye Movement Study

**DOI:** 10.1371/journal.pone.0030645

**Published:** 2012-02-27

**Authors:** Valentina Parma, Roberto Tirindelli, Angelo Bisazza, Stefano Massaccesi, Umberto Castiello

**Affiliations:** 1 Department of General Psychology, University of Padova, Padova, Italy; 2 Department of Neuroscience, University of Parma, Parma, Italy; Université de Toulouse, France

## Abstract

**Background:**

Evidence suggests that subliminal odorants influence human perception and behavior. It has been hypothesized that the human sex-steroid derived compound 4,16-androstadien-3-one (androstadienone) functions as a human chemosignal. The most intensively studied steroid compound, androstadienone is known to be biologically relevant since it seems to convey information about male mate quality to women. It is unclear if the effects of androstadienone are menstrual cycle related.

**Methodology/Principal Findings:**

In the first experiment, heterosexual women were exposed to androstadienone or a control compound and asked to view stimuli such as female faces, male faces and familiar objects while their eye movements were recorded. In the second experiment the same women were asked to rate the level of stimuli attractiveness following exposure to the study or control compound. The results indicated that women at high conception risk spent more time viewing the female than the male faces regardless of the compound administered. Women at a low conception risk exhibited a preference for female faces only following exposure to androstadienone.

**Conclusions/Significance:**

We contend that a woman's level of fertility influences her evaluation of potential competitors (e.g., faces of other women) during times critical for reproduction. Subliminally perceived odorants, such as androstadienone, might similarly enhance intrasexual competition strategies in women during fertility phases not critical for conception. These findings offer a substantial contribution to the current debate about the effects that subliminally perceived body odors might have on behavior.

## Introduction

The matter of subliminally perceived odors immediately calls up the topic of pheromones. Pheromones are chemical signals first discovered as sex attractants in animals, that appear to exert a behavioral or physiological response in animals of the same species [1]. As these chemicals have been primarily investigated in animals [2], in which the issue of awareness *per se* cannot be considered, the possibility that pheromones are also a human phenomenon and that they could have an effect on human behavior is currently being debated [3]–[9]. Due to the complexity of human behavior, efforts to measure behavioral modifications following exposure to putative pheromones have only been tentative [8], [10]. Some authors have, moreover, recently challenged the concept of pheromones in all species [9]. For all intents and purposes, the present study has circumvented that dispute and has concentrated its efforts on investigating if a subliminally perceived odorant can influence human behavior in view of the hypothesis that olfactory cues can mediate behavioral responses. Extensively studied with relation to its effects on mood, behavior and brain function [8], androstadienone, which is found in human sweat and other secretions, was the steroid compound investigated in the present study [11]–[14].

Although the issue is a highly disputed one [6], androstadienone, a member of the family of odorous 16-androstenes, is considered a putative male human pheromone. Androstadienone has not, however, always been detected in human secretions [14] and the gender differences reported may have been due to the small sample sizes assessed [10]. Although the physiological concentrations produced by human males and females has not been fully clarified [15]–[16], androstadienone has been experimentally administered at concentrations over a million times higher than levels naturally reported in the human body [17]. The chemical has been shown to have strong pheromone-like characteristics in the literature which has reported that androstadienone influences emotional attention, modulating interpersonal perception [18]–[20], increases women's positive and decreases negative moods in a context-dependent manner, enhances the feeling of being focused [17]–[19], [21]–[23], and increases women's tolerance to pain [24]. High doses of androstadienone, moreover, determine measurable changes in endocrine status [25] and autonomic arousal which appear to be specific to women [10], [18]–[19], [26]–[31]. In neural terms, there is evidence that the effect of androstadienone in women goes beyond the olfactory system determining activation of brain areas associated with attention, social cognition, and sexual behavior [32]–[37].

Considerable evidence seems to suggest that androstadienone might have a social function in that it may elicit behavioral responses in women by modulating mate-choice decisions. This hypothesis has been recently supported by a study carried out on speed-dating (ecological) situations. Although depending on the specific speed-dating situation, men were rated more attractive by women exposed to androstadienone compared to those exposed to a control compound [38]. In addition, the fact that women showed concordant strength of preference for facial masculinity and for the odor of androstadienone has been interpreted as indirect evidence that the chemical indexes male mate quality [39]. It is well known that mate preferences are strongly influenced by morphological traits [40]–[41] that might enhance physical attractiveness [42] probably because they signal mate genetic quality [43]. Interestingly, women near peak fertility time show a higher preference for visual (and auditory) masculinity and a lower one when their conception risk is low [44]–[47]. Consistent with evidence that androstadienone enhances women's preference for male faces and that the preference is naturally more pronounced when they are at high conception risk, we hypothesize that there is a link between conception risk and exposure to androstadienone. Does androstadienone's effect on women's attraction to men vary depending on the phase in their menstrual cycle? This is the question we have attempted to answer here.

## Experiment 1

### The Effect of Androstadienone on Eye Fixation Towards Female and Male faces and objects

Eye fixation times on female and male faces and on ordinary objects by heterosexual women at high conception risk (HCR, i.e. follicular phase) or low conception risk (LCR, i.e. luteal phase) times were recorded and measured.

### Materials and Methods

#### Ethics statement

The study's experimental procedures were approved by the Institutional Review Board at the University of Padova and were in accordance with the principles of the Declaration of Helsinki [48]. All the participants gave written consent to participate in the research.

#### Participants

Seated in separate areas to ensure privacy, the members of the original study cohort of 305 individuals (194 women and 111 men) were asked to fill out a questionnaire concerning their history of nasal congestion or infections, olfactory dysfunctions, and use of tobacco products or antidepressants. In the absence of direct hormone values, the questionnaire posed questions to the women about: use of hormone therapy, including oral or intravenous contraceptives during the precedent six months, the regularity of their menstrual cycle, and the possibility that they could be pregnant. Although all members of the original cohort took part in the study, data from the 23 women using contraceptives, the 36 with irregular menstrual cycle, the 8 who described themselves as homosexual or bisexual, the 2 who were possibly pregnant and the 15 who had used hormonal contraceptives during the 6 months before experimentation were not included in the analyses. Data from 16 participants (7 women and 9 men) were also excluded for technical reasons. As a result, the data from a total of 103 women (age: mean 22.6±1.0 years) and 102 men (age: mean 22±1.2 years) were included in the final analyses. To demonstrate that the effects of androstadienone are unique to females, male participants were included in the study for comparative purposes. Selection criteria were determined on the bases of the questionnaire administered prior to the experimental session.

Consistent with other studies in the literature, a cycle-length standardization formula was calculated to determine each woman's phase in her cycle. Although intra- and inter-women menstrual cycle variability is quite high, this is particularly true for the follicular rather than for the luteal phase [49]. We standardized the cycle length to a 28-day cycle by adjusting the follicular phase of each woman with respect to the length of her normal menstrual cycle. This process resulted in an invariant luteal phase which remained constant at 14 days. If a women was in her luteal phase, that is during the last 14 days of her cycle, her standardized cycle day was calculated by subtracting 28 days from the normal length of her cycle and adding the number counting from the first day of her cycle. Thus, if a woman had a 31-day cycle and it was the 23rd since the beginning of her cycle, her standardized cycle day would be calculated as follows: 28−31+23 = 20. If, instead, a woman was in her follicular phase, that is during the first 14 days of her cycle, her standardized cycle day was calculated by dividing her actual cycle day by the length of her normal cycle minus 14, multiplied by 14. If it was 10th day of her period and her cycle was normally 34 days long, then the standardized-cycle-day would be calculated as follows: [10/(34−14)]*14 = 7. For further details, refer to Garver-Apgar and colleagues [50].

On the basis of these calculations then, our sample was composed of 51 women in the HCR (or follicular) phase and 52 women in the LCR (or luteal) phase. Three experimental groups of participants (26 women HCR phase; 26 women LCR phase; 26 men) and three control groups were composed (25 women HCR phase; 26 women LCR phase; 26 men). Participants were not given information concerning they study's hypotheses or the identity of the compounds involved before the experiment was carried out. When debriefed and asked to describe the odor they had smelled, all the participants reported smelling a clove or spicy odor and none thought they had smelled odorants connected to the human body, confirming the implicit nature of the study results. As the chemical's odor was masked by clove oil, the risk of including super smellers [51], specific to the female groups since women are known to be generally better than men at odor task performances [52]–[53], was greatly reduced. A further test – sniffing a pure androstadienone solution - was also carried out in order to exclude the presence of participants selectively unable to smell androstadienone (i.e., specific anosmia to androstadienone). The results showed that none of the participants included within the final sample could be considered functionally anosmic to the experimental compound. Those participants (N = 27) resulting anosmic to androstadienone were excluded from the final analysis for other reasons.

#### Compounds

The experimental compound consisted of a 250 µM solution of 4,16-androstadien-3-one (Steraloids Inc., Newport, RI; purity ≥98%) dissolved in propylene glycol (purity ≥99%) containing 1% clove oil as an odor mask. The control compound consisted of 1% clove oil in propylene glycol. Measured amounts of solutions were pipetted onto pads which was used to apply the experimental or control compound to the area of the skin between the mouth and the nose of the participants. Approximately 2 nmols of androstadienone were applied to the epidermal surface of each of the participants. The methods and concentrations utilized were chosen to permit comparisons with findings already outlined in the literature [17], [20], [22]–[23], [32], [38]. Given that the concentration utilized is much higher than that usually found in nature [21] and quite near the olfactory threshold, [51] clove oil was added to mask thee conscious perception of its scent. The compounds were prepared by an independent chemist who revealed the code to the other experimenters only after the statistical analyses were completed.

#### Stimuli

**Figure 1:**
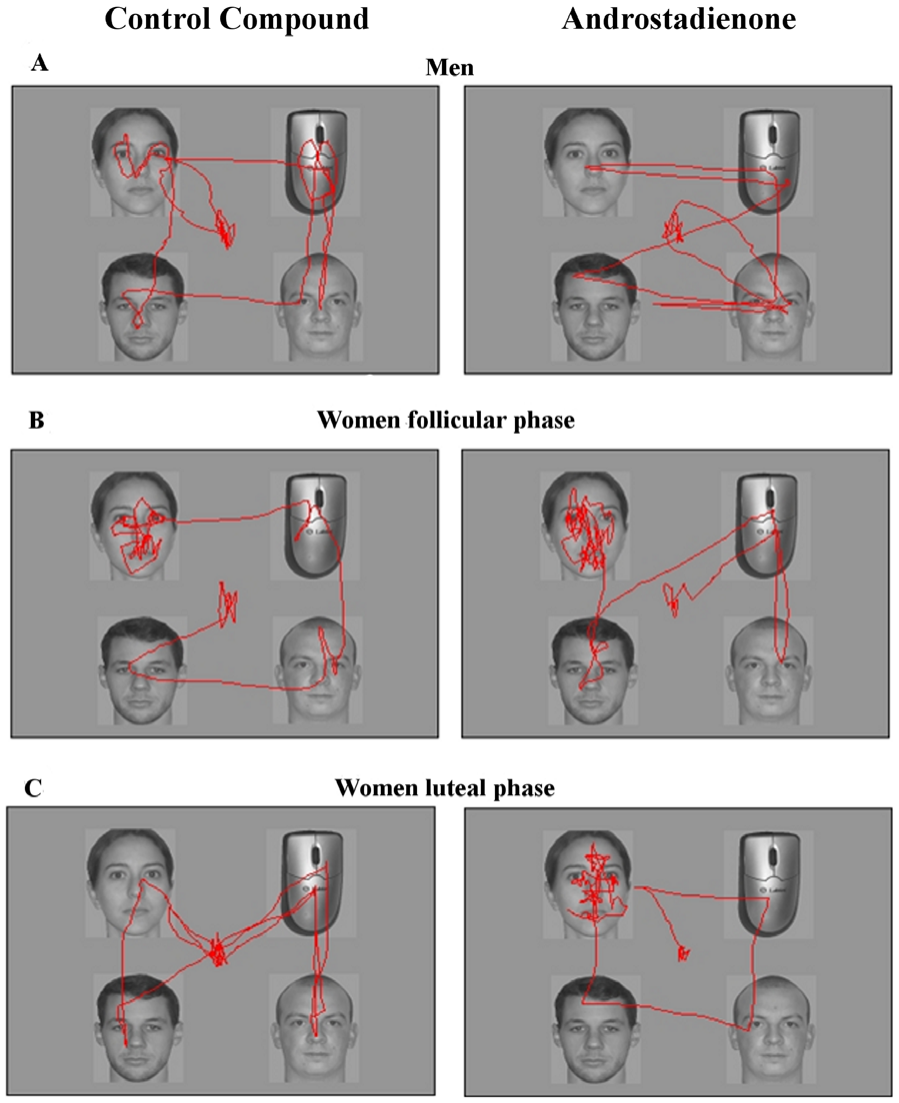
An example of eye movement trajectories. Eye movement trajectories following exposure to the control compound or androstadienone for (A) men, (B) women in the HCR phase and (C) women in the LCR phase.

The stimuli were arrays of four black and white photographs representing female (N = 12) and male (N = 12) faces, and familiar objects (N = 12) (Figure 1). The order of stimuli presentation was fully randomized between and within subjects. The items included within each stimulus together with their position within the array were varied at each trial. The items were included within a matrix in which it was possible to randomly locate four stimuli at fixed x, y coordinates: top-left area (180, 50 and 428, 362 pixels), top-right (597, 50 and 846, 362 pixels), bottom-left (180, 408 and 428, 718 pixels) and bottom-right (597, 408 and 846, 718 pixels). The photographs were standardized at 248×310 pixels maintaining the original proportions using an *ad-hoc* software (Paint Shop Pro, The Gimp). The photographs were of faces depicting ordinary looking persons between 20 and 30 age without distinctive signs (e.g., beard, piercing, particularly long hair). For standardization purposes each model was asked to maintain a neutral expression with his/her mouth closed and to remove any accessories (jewellery and eyeglasses). The photos of familiar objects included: a cigarette lighter, a screwdriver, a computer mouse, a telephone, a mobile telephone, a spoon, a highlighter, a glove, a light bulb, a pair of eyeglasses, a pair of scissors, a stapler and a pencil sharpener.

#### Eye-Tracking Measurement

The Eye Position Detector System (EPDS; sampling frequency: 40 ms) [54] was utilized to track eye movements. The EPDS gives two types of responses: a series of x–y coordinates corresponding to the sequence of points viewed by the participants on the stimulus and a visual pattern, i.e., the eye track pattern for each item. The computer screen was divided into four main areas of interest within which the items composing the stimulus array were presented. Those areas were considered the valid viewing points and the times the participants fixated on those points were registered and summed.

#### Procedures

After the participants filled out the questionnaire and signed the informed consent statement, they were accompanied to the testing room and seated in front of a computer monitor at a distance of ∼70 cm. Left alone in the testing room, they were monitored from an adjacent area via a mirror and video monitor. For the ‘eye movement’ task the participants were asked to view the stimulus for 1800 ms displayed on the computer monitor during which time eye movements were recorded. Each participant viewed four blocks of nine trials (N = 36). The experimental session lasted ∼60 min (from the time the participant arrived to the time he/she left). The room temperature and humidity was kept constant during the testing sessions.

#### Data Analysis

A three-way ANOVA with treatment (androstadienone, control compound) and group (women HCR phase, women LCR phase, men) as between-subjects factors and item category (females' faces, males' faces and objects) as within-subject factor was performed on the time spent by participants to view the different items composing the stimulus. Post hoc pairwise comparisons were performed using *t*-tests and Bonferroni's corrections were applied. A significance threshold of *P*<.05 was set for all statistical tests.

### Results

#### Qualitative Analysis of Eye Movement Trajectories

As shown in Figure 1*a*, the eye movement trajectory patterns of the male participants were not affected by exposure to the experimental or control compounds. The HCR-phase women spent more time viewing the female faces with respect to the male faces or the objects following exposure to both compounds (Figure 1*b*). The LCR-phase women showed the same eye movement trajectory pattern as the men following exposure to the control compound. When exposed to androstadienone, the LCR-phase women had a pattern that was similar to that observed in the HCR-phase women (Figure 1*c*) showing a marked interest in females faces with respect to the male faces and the objects (Figure 1*c*).

#### Viewing Time

**Figure 2:**
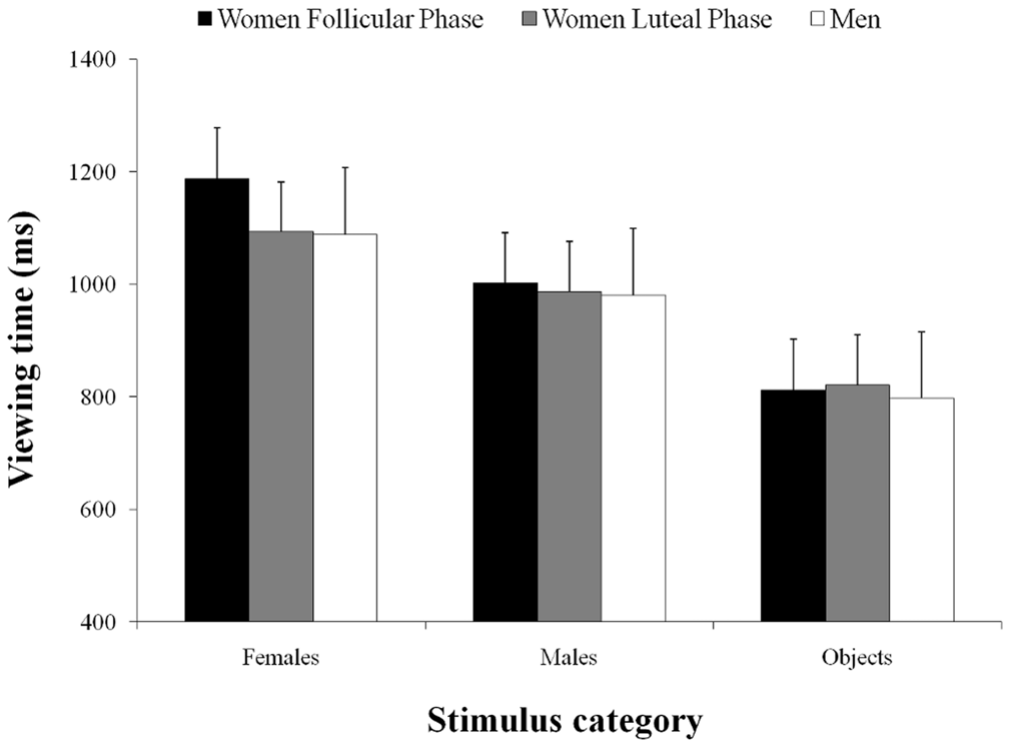
A graphical representation of the groups by item category. Mean viewing time in the three groups of participants with respect to each category. Bars represent the standard error of means.

**Figure 3:**
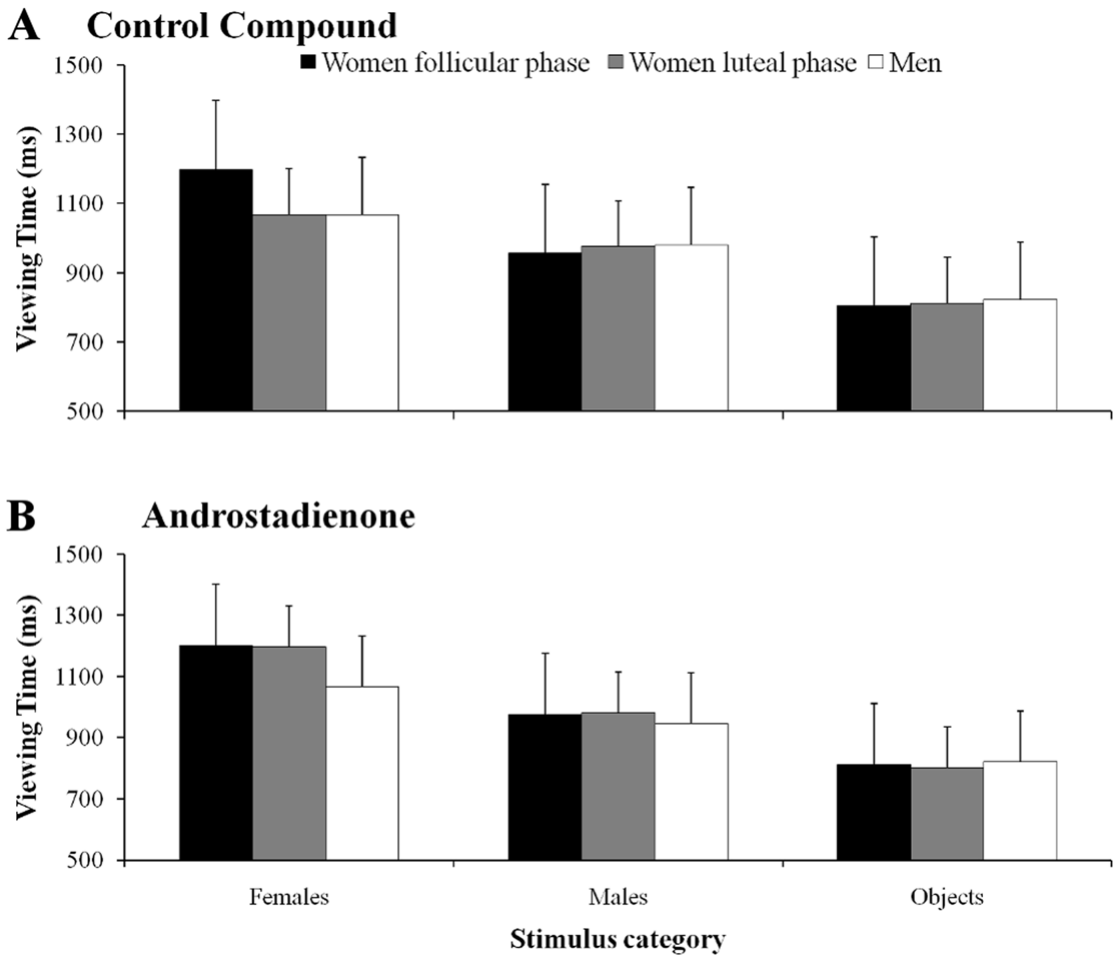
A graphical representation of the groups by category. Mean viewing time in the three groups of participants with respect to each category following exposure to the control compound (panel *a*) and to androstadienone (panel *b*). Bars represent the standard error of means.

Differences in viewing the various stimuli were significant (*F*_2,288_ = 52.39, *P*<0.0001, η^2^ = 0.83). Time spent viewing the female faces was longer with respect those spent viewing the male faces and the objects (1133 ms, 970 ms and 813 ms, respectively; *p_s_*<0.05). The comparison between the times spent viewing the male faces and the objects was also significant (*p*<0.05). The differences between groups were significant (*F*_4,288_ = 25.07, *P*<0.000, η^2^ = 0.66). Post-hoc contrasts revealed that all three groups spent more time viewing female faces with respect to the male faces and the objects (*P_s_*<0.05; see Figure 2). Time spent viewing the objects was similar across the groups (*P_s_*>0.05; Figure 2). The HCR-phase women spent more time viewing female faces than did the LCR-phase women or the men (*P_s_*<0.05; Figure 2). Analysis of the intergroup differences with regard to the various stimuli (*F*_4,288_ = 18.64, *P*<0.001, η^2^ = 0.63) indicated that when exposed to the control compound, all the groups spent more time viewing the female faces with respect to the males faces or the objects (*P_s_*<0.05; Figure 3*a*). The HCR-phase women spent more time viewing female faces than did the other two groups (*P_s_*<0.05; Figure 3*a*). Time spent viewing the objects was similar across the groups (*P_s_*>0.05; Figure 3*a*). Following exposure to androstadienone, the results were similar to those found following exposure to the control compound as far as the HCR-phase women and the men were concerned (Figure 3*b*). Exposure to the compound, however, provoked a change in the LCR-phase women who spent more time viewing the female faces with respect to the male faces and the objects (*P_s_*<0.05; Figure 3*b*). In that group, time spent viewing females faces after exposure to androstadienone was longer than that spent following exposure to the control compound (1198 vs. 1068 ms; *p*<0.05). All the remaining differences across groups, with regard to the type of compound used, and the stimuli were not significant (*P_s_*>0.05; Figure 3*a–b*).

We had originally hypothesized a link between conception risk and exposure to androstadienone and specifically that the chemical influenced women's attraction for male faces depending on their menstrual cycle phase. The results presented here indicate that androstadienone does modulate women's reaction to facial stimuli depending on their menstrual cycle phase, but that the modulation is connected to female rather than to male stimuli. Specifically, LCR-phase women exposed to androstadienone spent more time viewing female faces than when they were exposed to the control compound. This effect, together with the result that HCR-phase women spent more time viewing female faces regardless of the compound administered seems to provide indirect evidence that androstadienone can trigger intrasexual competition strategies by which members of the same sex compete for mating access to members of the opposite sex [55].

Intrasexual competition has recently been investigated by assessing the influence of fertility on the score women give to photographs of male and female faces [56]. Specifically, derogation - any act intended to decrease a rival's perceived value - was the competitive strategy that was studied. It was found that during high fertility periods, competition, and hence derogation, was stronger and this was confirmed by lower ratings with regard to female facial attractiveness. Consistent with these observations, the present results seem to indicate that presumably because they need to evaluate potential rivals at a time critical to select a mate, the HCR-phase women tend to pay more attention to other women than to men regardless of the compound administered while the LCR-phase women behave this way only when they are exposed to androstadienone.

Subjective scores of visual stimuli were thus collected to assess intrasexual competition.

## Experiment 2

Investigations indicating that female faces are rated significantly more attractive than male faces [57], [58] suggest that female attractiveness is of evolutionary importance, and hence, a potential vehicle for competition. The present experiment assessing intrasexual competition with regard to attractiveness replicated that presented by Fisher [56]. Following exposure to the control compound, or androstadienone, the participants were asked to rate the facial attractiveness of the female faces utilized in experiment 1. On the basis of the results obtained in experiment 1, we expected the HCR-phase women to rate female faces less attractive with respect to the LCR-phase women following exposure to both compounds and that the latter group would rate the female faces less attractive only after exposure to androstadienone.

### Materials and Methods

#### Participants

All 103 women who took part in experiment 1 also participated in the present study and did so during the same menstrual phase as when they participated in the previous one. Care was taken to ascertain that they still met all the recruitment criteria and that the conditions outlined in the questionnaire were invariable with respect to the time the first experiment was carried. As in the previous experiment, there were 51 HCR-phase women (days 6–15) and 52 LCR-phase women (days 0–5 and days 16–28). The participants were randomly assigned to two experimental groups exposed to androstadienone (26 women HCR phase; 26 women LCR phase) and two control groups exposed to the control compound (25 women HCR phase; 26 women LCR phase). As no effects were detected in male participants in experiment 1, none were included in the second experiment. For similar reasons the ‘object’ category was also excluded.

#### Procedures

Participants were asked to rate the facial attractiveness of each of the female faces used for the ‘eye movement’ experiment using a Likert-type scale (1 = extremely unattractive to 7 = extremely attractive). Each of the faces used in experiment 1 was presented at the center of a computer screen.

#### Data Analysis

An ANOVA was used to test for differences between groups (women HCR phase, women LCR phase) and compounds (androstadienone, control). Post hoc contrasts using *t*-tests and Bonferroni's corrections were applied. A significance threshold of *P*<.05 was set for all statistical tests.

### Results

**Figure 4:**
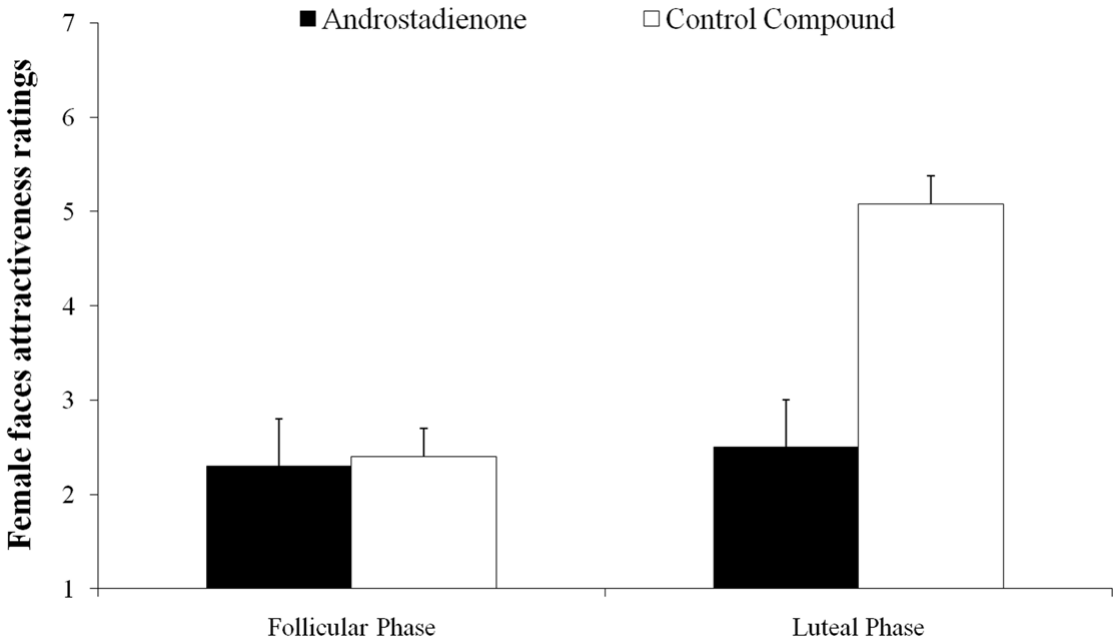
A graphical representation of the ratings of facial attractiveness by the two groups. Mean ratings of facial attractiveness for the LCR- phase and HCR- phase women following exposure to the control compound or androstadienone. Bars represent the standard error of means.

The interaction ‘group by compound’ was found to be significant (*F*_1,96_ = 27.32, *P*<0.0001, η^2^ = 0.71). Post-hoc tests revealed that the HCR-phase women exposed to the control compound rated females' faces significantly less attractive with respect to the LCR-phase women (*P*<0.05; Figure 4). No differences were found in the ratings given by the HCR-phase women following exposure to the two compounds (*P*>0.05; Figure 4). The LCR-phase women gave different ratings depending on the compound administered and rated females faces less attractive following exposure to androstadienone (*P*<0.05). These findings corroborate those obtained in experiment 1 and support the hypothesis that female intrasexual competition, in particular through competitor derogation, are affected by subliminal odorants such as androstadienone.

## Discussion

The aim of this study was to investigate how androstadienone affects women's judgment on facial stimuli during different phases of their menstrual cycle on. Generally speaking, female faces were viewed for longer times than were male faces or objects. The HCR-phase women concentrated greater attention on female faces than did the LCR-phase women regardless of odorant exposure. The LCR-phase women spent more time viewing female faces following androstadienone exposure.

Given the numerous studies that have reported an increased preference for masculinity during ovulation [45] and following exposure to androstadienone [38], these results might appear surprising. We did not, in fact, expect to find differences in the ratings of and behavioral response to female facial stimuli depending on the compounds administered and the phase in the women's menstrual cycle. Since no studies, with the exception of Hummer and McClintock's [20], have examined the link between conception risk and androstadienone exposure in the arena of facial processing, the present findings can be seen as a novel addition to this body of research.

In contrast to previous research on a similar topic, photographs of real people rather than variations on computer-generated facial stimuli [39], [44]–[46] were utilized here. The argument has been made, in fact, that it might not be a reliable assumption to evaluate preferences during the menstrual cycle using morphed faces - which might “translate to actual female choice” (pp. 2) [59] - as unmanipulated photographs might convey them better since they are closer to real world situations.

Eye movements, instead of self-reported measures [60], were used as the former are not dependent on explicit processes and can provide a precise measure of the duration of visual interest [61]. Although previous evidence suggests that androstadienone enhances feelings of being focused [17], [22], no studies have reported on its effect on sustained attention [23]. The recently published work by Hummer and McClintock [20] demonstrated that androstadienone may be able to increase attention to different forms of emotional stimuli within an environment. Following this train of thought connected to a number of psychological effects related to androstadienone exposure [21], [24], it is plausible that a chemosensory attention related effect does exist and can be tested in a biologically relevant framework (e.g. in the context of mate selection process) but not in laboratory conditions [23]. It has been shown, in fact, that female response to androstadienone is reflected in brain areas involved in sustained attention [34].

Although all the female and male facial stimuli used in the present experiments presented neutral expressions, the results suggest that those stimuli may have prompted an emotional response drawing the participants' attention. But, why did HCR-phase women spend more time viewing female faces and rating them as less attractive regardless of the compound administered? And, on the contrary, why did the LCR-phase women manifest similar behavior only when exposed to androstadienone? It can be hypothesized that the effects can be related to derogation, an intrasexual competition strategy used to decrease a rival's value [62]. It appears to be a particularly relevant strategy for women who find themselves competing for desirable mates who must provide resources rather than attractiveness [55].

But who can be considered a “rival” in our experimental model? A number of studies investigating how androstadienone modulates moods indicated the importance of different contexts. As an example, Jacob and colleagues [27] noted that women exposed to androstadienone rated their mood more positively in the presence of a male rather than a female experimenter. It is possible then that a female technician, as in our case, could have triggered the competition. In addition, consistent with Zajonc's affective primacy theory [63], it is possible that sensory inputs (either conscious or subliminally perceived) requiring minimal cognitive involvement (such as receiving task instructions from a female experimenter or smelling a masked body odor) might play a determinant role in human reproductive biology [64].

The fact that both groups of women show this ‘competitive’ pattern following androstadienone exposure seems to confirm the relevance of the compound. Mazzatenta and colleagues [31] recently hypothesized that androstadienone could have two-fold “pheromonal-like” characteristics in high-peak fertility women. As reflected in facial thermal skin fluctuations, androstadienone might first act as a releaser, triggering a sexual arousal status - which may have set off competition in our case- and secondly as a modulator – affecting the eye movement pattern registered. In this respect, these findings seem to be consistent with those reported in a previous study which demonstrated female intrasexual competition in terms of attractiveness [56]. Specifically, during critical times for reproduction women were found to be more derogatory of female facial attractiveness compared to infertile phases. Analogous findings were found in HCR-phase women using the same methodology. Using a more implicit method, we were able to ascertain that participants viewed female with respect to male faces longer and with greater attention. Moreover, in contrast to a previously published study [65], we provided evidence that the ratings women participants gave to female attractiveness were modulated when they were exposed to a biologically relevant odorant at least during a low fertility phase.

On the basis of our and others' findings, it would seem that androstadienone modulates voluntary eye movements by eliciting a cascade of physiological and psychological events. With a certain degree of caution, it can also be hypothesized that exposure to below threshold quantities of the chemical masked by another odor had an effect on our participants' endocrine system, as previously shown [25]. This would be consistent with evidence that in some situations its exposure can lead to measurable alterations in the endocrine system which in turn might modulate sexual strategies [25]. It is unclear why androstadienone does not provoke cumulative effects in terms of female facial processing in highly fertile women. It is possible that the effects of androstadienone in these women are prevented to avoid non-adaptive behaviors, such as the degeneration of derogation in manifest verbal (or physical) aggression towards same-sex rivals [66].

There were some limitations to the present study. First, the fact that female judgment was confined to the attractiveness of females' faces could have been extended to evaluate emotional dimensions such as fear and anger which could reinforce the level of intrasexual competition. This aspect could be considered in future experiments in which the emotional content of facial expression is manipulated. Second, the design did not allow for any examination of the effects of androstadienone to a specific intrasexual competition behavior. Given that there are several ways in which women compete intrasexually (e.g., derogating attractiveness, derogating other women's fidelity) further research is required to determine if the chemical's effect extends to all or only some vehicles of competition. Third, we were unable to control for relationship status. Some investigations on intrasexual strategies have indicated that tactics for competitor derogation are influenced by the expected duration of the relationship [67]. It is possible that the strength of intrasexual competition varies in terms of the desired relationship duration and feelings of commitments. Fourth, this line of research might benefit from the use of solutions in which the concentration of androstadienone used is consistent with that naturally present in the environment. Fifth, using different odorous body secretions (e.g. isovaleric acid) as control compounds might give further insights in separating the specific effects produced by androstadienone from those provoked by biologically relevant odors in general. Finally, future studies should include use of objective measurements (e.g. luteinizing hormone detection kit) instead of self-reports concerning women's menstrual cycle in order to reduce possible hormonal variations influencing sensory and judgment abilities on topics related to mate choice. Finally, no hormone testing was carried out. As suggested by Wyart and colleagues [25], future investigations may be able to reveal a possible correlation between changes in endocrine state following exposure to androstadienone and differential behavioral responses to the compound throughout the menstrual cycle.

In conclusion, our results present a novel contribution suggesting that women's level of fertility influences the assessment of potential competitors, causing them to view the faces of other women more attentively at times critical for reproduction. On the basis of these findings, subliminally perceived odorants such as androstadienone seem to be able to enhance intrasexual competition strategies in women during fertility phases not critical for conception.
